# Synthesis and antimicrobial evaluation of two peptide LyeTx I derivatives modified with the chelating agent HYNIC for radiolabeling with technetium-99m

**DOI:** 10.1186/s40409-016-0070-y

**Published:** 2016-04-22

**Authors:** Leonardo Lima Fuscaldi, Daniel Moreira dos Santos, Natália Gabriela Silva Pinheiro, Raquel Silva Araújo, André Luís Branco de Barros, Jarbas Magalhães Resende, Simone Odília Antunes Fernandes, Maria Elena de Lima, Valbert Nascimento Cardoso

**Affiliations:** Department of Clinical and Toxicological Analyses, School of Pharmacy, Federal University of Minas Gerais, Av. Antônio Carlos, 6627, Belo Horizonte, MG 31270-901 Brazil; Department of Biochemistry and Immunology, Institute of Biological Sciences, Federal University of Minas Gerais, Belo Horizonte, MG Brazil; Department of Chemistry, Institute of Exact Sciences, Federal University of Minas Gerais, Belo Horizonte, MG Brazil

**Keywords:** Septic and aseptic inflammation, Differential diagnosis, Antimicrobial peptides, LyeTx I derivatives, MALDI-ToF-MS, RP-HPLC, Technetium-99m, HYNIC, EDDA, Tricine

## Abstract

**Background:**

Current diagnostic methods and imaging techniques are not able to differentiate septic and aseptic inflammation. Thus, reliable methods are sought to provide this distinction and scintigraphic imaging is an interesting option, since it is based on physiological changes. In this context, radiolabeled antimicrobial peptides have been investigated as they accumulate in infectious sites instead of aseptic inflammation. The peptide LyeTx I, from the venom of *Lycosa erythrognatha*, has potent antimicrobial activity. Therefore, this study aimed to synthesize LyeTx I derivatives with the chelating compound HYNIC, to evaluate their antimicrobial activity and to radiolabel them with ^99m^Tc.

**Methods:**

Two LyeTx I derivatives, HYNIC-LyeTx I (N-terminal modification) and LyeTx I-K-HYNIC (C-terminal modification), were synthesized by Fmoc strategy and purified by RP-HPLC. The purified products were assessed by RP-HPLC and MALDI-ToF-MS analysis. Microbiological assays were performed against *S. aureus* (ATCC® 6538) and *E. coli* (ATCC® 10536) in liquid medium to calculate the MIC. The radiolabeling procedure of LyeTx I-K-HYNIC with ^99m^Tc was performed in the presence of co-ligands (tricine and EDDA) and reducing agent (SnCl_2_^**.**^2H_2_O), and standardized taking into account the amount of peptide, reducing agent, pH and heating. Radiochemical purity analysis was performed by thin-layer chromatography on silica gel strips and the radiolabeled compound was assessed by RP-HPLC and radioactivity measurement of the collected fractions. Data were analyzed by ANOVA, followed by Tukey test (*p*-values < 0.05).

**Results:**

Both LyeTx I derivatives were suitably synthesized and purified, as shown by RP-HPLC and MALDI-ToF-MS analysis. The microbiological test showed that HYNIC-LyeTx I (N-terminal modification) did not inhibit bacterial growth, whereas LyeTx I-K-HYNIC (C-terminal modification) showed a MIC of 5.05 μmol^.^L^−1^ (*S. aureus*) and 10.10 μmol^.^L^−1^ (*E. coli*). Thus, only the latter was radiolabeled with ^99m^Tc. The radiochemical purity analysis of LyeTx I-K-HYNIC-^99m^Tc showed that the optimal radiolabeling conditions (10 μg of LyeTx I-K-HYNIC; 250 μg of SnCl_2_^**.**^2H_2_O; pH = 7; heating for 15 min) yielded a radiochemical purity of 87 ± 1 % (*n* = 3). However, RP-HPLC data suggested ^99m^Tc transchelation from LyeTx I-K-HYNIC to the co-ligands (tricine and EDDA).

**Conclusions:**

The binding of HYNIC to the N-terminal portion of LyeTx I seems to affect its activity against bacteria. Nevertheless, the radiolabeling of the C-terminal derivative, LyeTx I-K-HYNIC, must be better investigated to optimize the radiolabeled compound, in order to use it as a specific imaging agent to distinguish septic and aseptic inflammation.

## Background

Inflammatory processes can be divided into two categories: septic (induced by bacteria or fungi) or aseptic (absence of microorganisms) inflammation [1]. Thus, differential diagnosis is required to determine the most suitable therapeutic approach. In some cases, such as bone inflammation, current diagnostic methods and conventional imaging techniques are not able to differentiate septic and aseptic inflammation. Therefore, alternatives must be found in order to ensure an accurate diagnosis. In this sense, scintigraphic imaging is a promising approach since it is based on physiological changes, which occur earlier than anatomical modifications [2–4]. In this context, radiolabeled antimicrobial peptides have been investigated as possible suitable tools, since they accumulate in infectious sites instead of in aseptic inflammatory lesions, once they preferentially bind to bacteria and fungi [5–7].

The cationic peptide LyeTx I was primarily isolated from *Lycosa erythrognatha* venom. After its purification and characterization, it was obtained by chemical synthesis. The peptide is composed of 25 amino acid residues and carries a natural carboxyl-terminal (C-terminal) carboxyamide (H-IWLTALKFLGKNLGKHLAKQQLAKL-NH_2_). LyeTx I exhibits antimicrobial activity against microorganisms, such as *Escherichia coli*, *Staphylococcus aureus, Candida krusei* and *Cryptococcus neoformans* [8]. Therefore, radioisotope-labeled LyeTx I may be an interesting strategy for a specific imaging probe for infections.

The radioisotope technetium-99m (^99m^Tc) presents suitable features for its administration to patients in nuclear medicine. This radionuclide emits gamma rays of low energy (~140 keV) and has physical half-life of 6.02 h. ^99m^Tc exposes patients to low radiation doses whereas it is widely used for radiolabeling molecules employed as scintigraphic imaging probes. Furthermore, this radioisotope is easily obtained from a low cost molibdenium-99/technetium-99m (^99^Mo/^99m^Tc) generator [9, 10]. However, in order to use ^99m^Tc for radiolabeling peptides without disulfide bonds, such as LyeTx I, it is necessary to attach a chelating agent to the amino acid sequence. In this sense, 2-hydrazinonicotinamide (HYNIC) is a good option, since its carboxylic acid group reacts directly with the nitrogen-terminal (N-terminal) residue or alternatively with the lateral amino group of a lysine residue present in the peptide sequence. However, an extra lysine may be coupled to the C-terminal portion in order to maintain the peptide sequence with a minor change. Lastly, to stabilize ^99m^Tc binding to HYNIC, tricine and ethylenediamine-N,N’-diacetic acid (EDDA) are used as co-ligands in the radiolabeling procedure [11–13].

Therefore, this study aimed to synthesize two peptide LyeTx I derivatives modified with the chelating agent HYNIC, to evaluate the maintenance of its antimicrobial activity and to standardize its radiolabeling with ^99m^Tc atoms.

## Methods

### Materials

Amino acid derivatives for peptide synthesis were purchased from Iris Biotech GmbH (Marktredwitz, Germany). Trifluoroacetic acid (TFA) and triisopropylsilane were obtained from Sigma-Aldrich (Saint Louis, USA). 1,3-diisopropylcarbodiimide was acquired from Fluka (Steinheim, Germany). 1-hydroxybenzotriazole was purchased from NovaBiochem-Merck (Darmstadt, Germany). N,N-dimethylformamide (DMF) and diisopropyl ether were obtained from Vetec (Duque de Caxias, Brazil). Acetonitrile (HPLC grade) was acquired from JT Baker (Center Valley, USA). If not mentioned otherwise, analytical grade solvents were used. All solvents used in reverse phase-high performance liquid chromatography (RP-HPLC) system (HPLC grade) were purchased from Tedia (Rio de Janeiro, Brazil). Ultrapure water, obtained through MilliQ® system of Millipore (Darmstadt, Germany), was used throughout. The bacterial strains of reference, *S. aureus* (ATCC® 6538) and *E. coli* (ATCC® 10536), were acquired from American Type Culture Collection – ATCC (Manassas, USA). ^99m^Tc was obtained from a ^99^Mo/^99m^Tc generator supplied by the Nuclear Energy Research Institute – IPEN (São Paulo, Brazil). Other reagents and solvents for the radiolabeling procedure were acquired from Sigma-Aldrich (São Paulo, Brazil).

### Synthesis and purification of two peptide LyeTx I derivatives modified with the chelating agent HYNIC

Two peptide LyeTx I derivatives with the chelating agent HYNIC attached either to its N-terminal residue (HYNIC-LyeTx I) or to its C-terminal portion (LyeTx I-K-HYNIC) were synthesized and purified, as previously reported [14].

Both synthesis were performed by stepwise solid-phase using the N-9-fluorenylmethyloxycarbonyl (Fmoc) strategy on a rink amide resin (0.63 mmol^**.**^g^−1^). Side chain protecting groups were as follows: *t*-butyl for threonine, *t*-butyloxycarbonyl for lysine and tryptophan, (triphenyl) methyl for histidine, asparagine and glutamine. Couplings were performed with 1,3-diisopropylcarbodiimide/1-hydroxybenzotriazole in DMF for 60–180 min. Deprotections (15 min, twice) were conducted by piperidine: DMF (1:4; v:v). Cleavage from the resin and final deprotection were performed with TFA/water/triisopropylsilane (95.0/2.5/2.5, v:v) at room temperature during 90 min. Post-precipitation of the products with cold diisopropyl ether, the crude peptide complexes were extracted with water:acetonitrile (1:1; v:v), followed by freeze-drying.

The crude synthetic products were purified by RP-HPLC on a C8 column (Discovery® BIO Wide Pore C8 column, 5 μm, 250.0 mm × 4.6 mm), previously equilibrated with 0.1 % (v:v) TFA in water (eluent A) and eluted by a linear gradient of 0.1 % (v:v) TFA in acetonitrile (eluent B), as specified in Table 1A.

**Table 1:** Solvent conditions for RP-HPLC

(A) Crude synthetic product purification		(B) Purified synthetic product analysis		(C) LyeTx I-K-HYNIC-^99m^Tc evaluation	
Time	Gradient of eluent B	Time	Gradient of eluent B	Time	Gradient of eluent B
(min)	(%)	(min)	(%)	(min)	(%)
0–8.2	0	0–3.7	0	0–5.0	0
8.2–12.4	0–30	3.7–33.5	0–100	5.0–30.0	0–55
12.4–50.0	30–55	33.5–39	100	30.0–35.0	55–100
50.0–54.0	55–100			35.0–45.0	100
54.0–62.5	100				

Flow = 1.0 mL^.^min^−1^. Detection = 214 nm

The collected fractions were assessed by matrix-assisted laser desorption ionization time of flight mass spectrometer (MALDI-ToF-MS) analysis on AutoFlex III (Bruker Daltonics®, Germany). Briefly, samples were spotted onto a sample plate (MTP 384 Anchorchip, Bruker Daltonics®, Germany) mixed with a saturated solution of α-cyano-4-hydroxycinnamic acid and allowed to dry at room temperature (dried-droplet method). The mass spectrometer (MS) spectra were acquired in the positive reflector mode with external calibration (Peptide Calibration Standard II, Bruker Daltonics®, Germany).

### Purity assessment of the peptide LyeTx I derivatives modified with the chelating agent HYNIC

The purified synthetic products were analyzed by RP-HPLC on a C18 analytical column (PepMap C18^TM^ column, 5 μm, 150.0 mm × 4.6 mm), previously equilibrated with 0.1 % (v:v) TFA in water (eluent A) and eluted by a linear gradient of 0.1 % (v:v) TFA in acetonitrile (eluent B), as specified in Table 1B. The peaks of the peptides were collected and analyzed by MALDI-ToF-MS on AutoFlex III (Bruker Daltonics®, Germany), as described in the previous section.

### In vitro evaluation of the maintenance of the antimicrobial activity of the peptide LyeTx I derivatives modified with the chelating agent HYNIC

The maintenance of the antimicrobial activity after peptide LyeTx I modifications with HYNIC was evaluated by microdilution test, according to the Clinical and Laboratory Standards Institute [15]. Bacterial strains of reference, *S. aureus* (ATCC® 6538) and *E. coli* (ATCC® 10536), were grown on tryptic soy agar at 37 °C for 18 h. Then, 0.5 McFarland scale bacterial suspensions (10^8^ CFU^.^mL^−1^) were prepared on tryptic soy broth (TSB). The readouts were carried by determination of minimum inhibitory concentration (MIC), defined as a reduction of 100 % in bacterial growth post-incubation with the peptide LyeTx I derivatives at 37 °C for 24 h. LyeTx I obtained by chemical synthesis and without the coupled chelating agent was used as treatment control. Only TSB (no bacterial suspension and no peptide) was used as negative control. TSB plus bacterial suspension (no peptide) were used as positive control. MIC was expressed as median (*n* = 3). Each replicate was performed with a different bacterial colony, in duplicate.

### Radiolabeling and radiochemical purity of LyeTx I-K-HYNIC with ^99m^Tc

The radiolabeling procedure of LyeTx I-K-HYNIC with ^99m^Tc and radiochemical purity analysis were performed as previously reported elsewhere [16], with some modifications. Briefly, in a sealed vial, tricine (20 mg) and EDDA (5 mg) were solubilized in 0.9 % NaCl (w:v) solution (200 μL). Next, LyeTx I-K-HYNIC (5, 10 or 20 μg) and 1 mg^.^mL^−1^ SnCl_2_^**.**^2H_2_O solution (100, 200, 250 or 300 μL) in 0.25 mol^.^L^−1^ HCl were added. Then, the pH was adjusted (5, 6, 7, 8 or 9). Finally, Na^99m^TcO_4_ (37 MBq; q.s. ad = 1000 μL) was added to the vial and the final solution was heated (100 °C) in water bath (5, 15 or 30 min) or not heated. Radiochemical purity analysis of LyeTx I-K-HYNIC-^99m^Tc was performed by thin-layer chromatography on silica gel strips (Merck®). Methyl ethyl ketone (MEK) and acetonitrile:water (1:1; v:v) were used to determine the amount of free technetium (^99m^TcO_4_^−^) and hydrolyzed technetium (^99m^TcO_2_), respectively. Radioactivity was measured using an automatic gamma counter (Wizard, Finland).

### LyeTx I-K-HYNIC-^99m^Tc evaluation

LyeTx I-K-HYNIC-^99m^Tc was evaluated as previously described [17], by RP-HPLC on a C8 column (ACE 5 C8 column, 5 μm, 250.0 mm × 4.6 mm), previously equilibrated with 0.1 % (v:v) TFA in water (eluent A) and eluted by a linear gradient of 0.1 % (v:v) TFA in acetonitrile (eluent B), as specified in Table 1C. LyeTx I-K-HYNIC, EDDA and tricine were separately injected and the detection was at 214 nm. LyeTx I-K-HYNIC-^99m^Tc was injected, the fractions were collected and the radioactivity was measured using an automatic gamma counter (Wizard, Finland).

### Statistical analysis

Quantitative data were expressed as mean ± standard deviation (SD). Means were compared using Analysis of Variance (ANOVA), followed by Tukey multiple comparisons test. *p-*values < 0.05 were considered significant. Data were analyzed using the Prism software (version 5.0).

## Results and discussion

### Synthesis, purification and purity assessment of two peptide LyeTx I derivatives modified with the chelating agent HYNIC

**Fig. 1:**
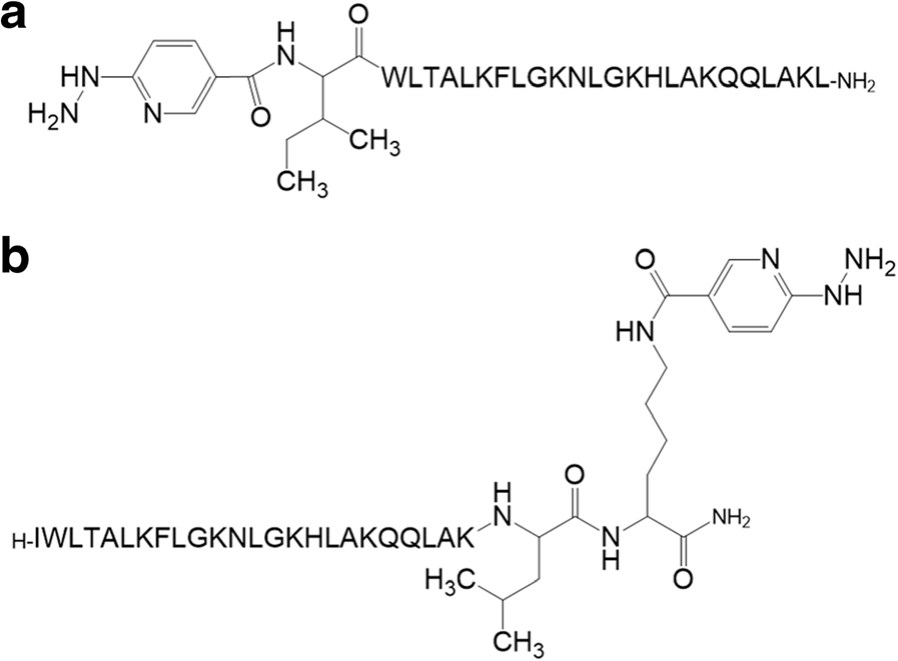
Structures of the peptide LyeTx I derivatives modified with the chelating agent HYNIC. **a** HYNIC-LyeTx I derivative (M_w_ = 2966 g^**.**^mol^−1^) and **b** LyeTx I-K-HYNIC derivative (M_w_ = 3094 g^**.**^mol^−1^). –NH_2_ represents the C-terminal carboxyamidation. H– represents the absence of N–terminal modification. M_w_: molecular weight

**Fig. 2:**
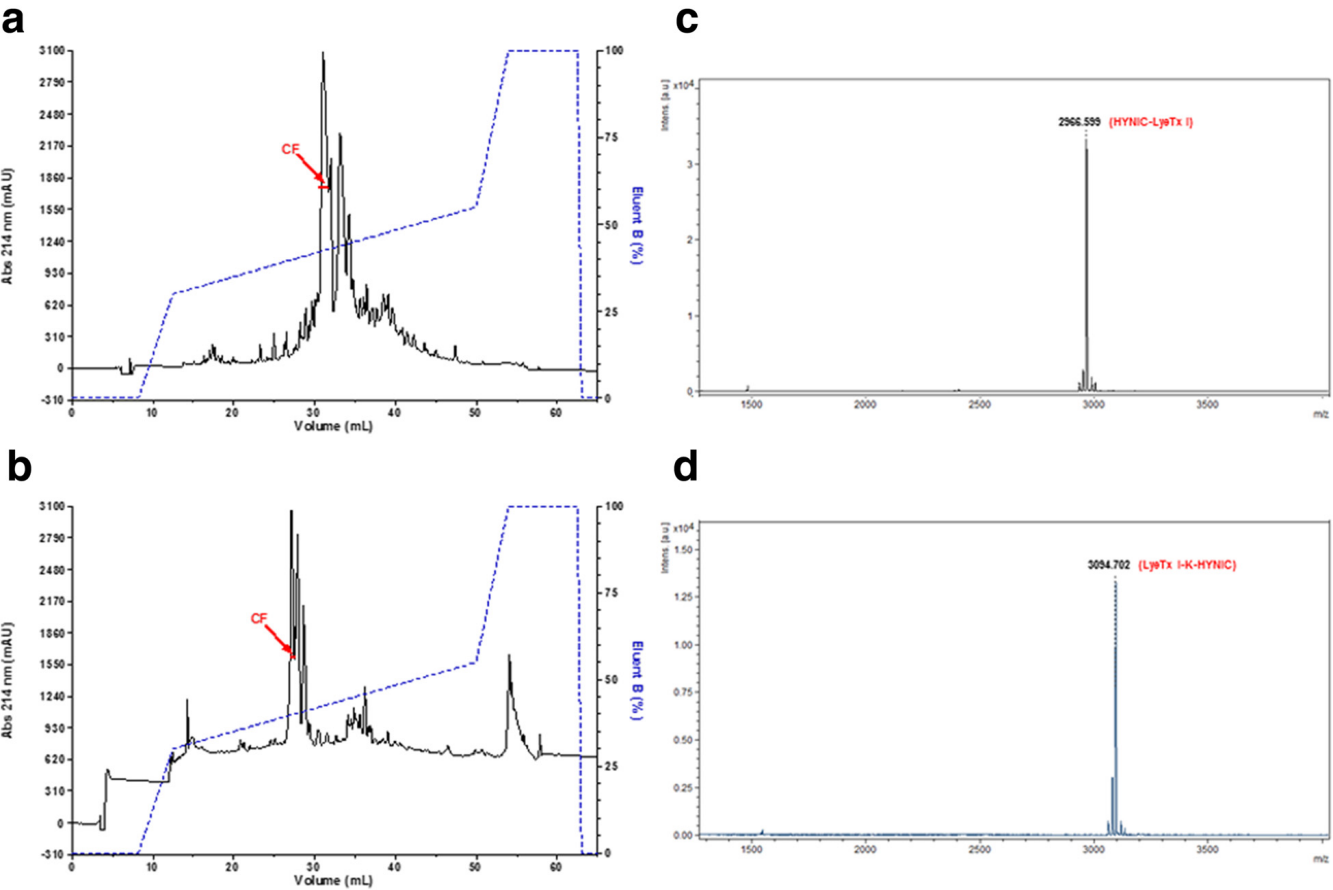
Purification of synthetic crude peptide LyeTx I derivatives modified with the chelating agent HYNIC, by RP-HPLC (Ettan LC, GE HealthCare, USA). Chromatograms of synthetic crude **a** HYNIC-LyeTx I and **b** LyeTx I-K-HYNIC: Discovery® BIO Wide Pore C8 column (5 μm, 250.0 mm × 4.6 mm) equilibrated with 0.1 % (v:v) TFA in water (eluent A) and eluted by a linear gradient of 0.1 % (v:v) TFA in acetonitrile (eluent B); the flow was 1.0 mL^**.**^min^−1^ and the detection was at 214 nm. Mass spectrometer (MS) spectra of the collected fraction (CF) of synthetic purified **c** HYNIC-LyeTx I and **d** LyeTx I-K-HYNIC: the molecular weights were 2966 Da and 3094 Da, respectively, obtained by deconvolution of the MS spectra

Two peptide LyeTx I derivatives were synthesized with the chelating agent HYNIC attached either to its N-terminal residue (Fig. 1a) or to the lateral amino group of an extra lysine residue coupled to its C-terminal portion (Fig. 1b). The synthetic crude products were purified by RP-HPLC (Fig. 2a, b) and the collected fractions were assessed by MALDI-ToF-MS analysis. Pure products with m/z 2966 (Fig. 2c) and m/z 3094 (Fig. 2d) were detected.

**Fig. 3:**
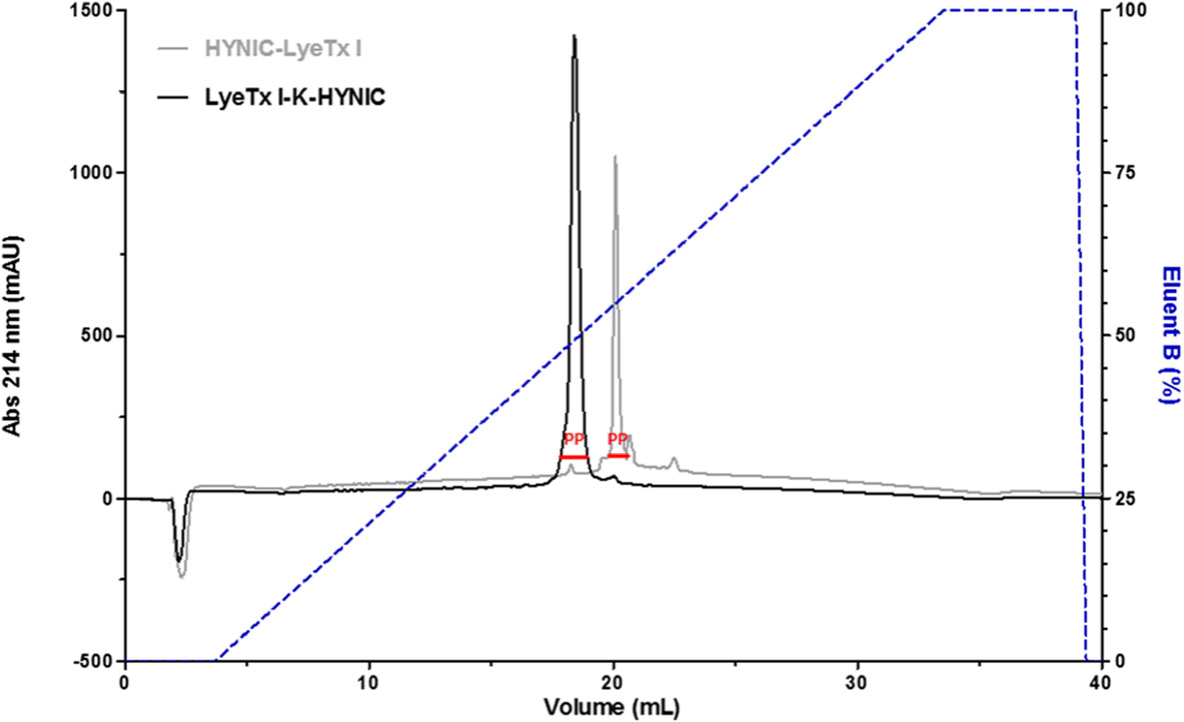
Purity assessment of synthetic purified peptide LyeTx I derivatives modified with the chelating agent HYNIC, by RP-HPLC (Ettan LC, GE HealthCare, USA). Chromatograms of synthetic purified HYNIC-LyeTx I (*gray line*) and LyeTx I-K-HYNIC (*black line*): PepMap C18^TM^ column (5 μm, 150.0 mm × 4.6 mm) equilibrated with 0.1 % (v:v) TFA in water (eluent A) and eluted by a linear gradient of 0.1 % (v:v) TFA in acetonitrile (eluent B); the flow was 1.0 mL^**.**^min^−1^ and the detection was at 214 nm. PP: peak of peptide

The synthetic pure products were assessed by RP-HPLC (Fig. 3). Both chromatograms exhibited single and well-defined peak of the respective peptide LyeTx I derivative in high purities: 93.36 ± 0.43 % (HYNIC-LyeTx I) and 97.13 ± 0.23 % (LyeTx I-K-HYNIC). Both peaks were collected and analyzed by MALDI-ToF-MS. Data showed similar MS spectra as those previously presented (Fig. 2c, d).

These findings indicated that both synthetic peptide LyeTx I derivatives were suitably synthesized and purified. Thus, the synthetic products were available for further evaluation of the antimicrobial activity.

### In vitro evaluation of the maintenance of the antimicrobial activity of the peptide LyeTx I derivatives modified with the chelating agent HYNIC

The maintenance of the antimicrobial activity after peptide LyeTx I chemical modifications was assessed by means of microdilution test, followed by incubation with *S. aureus* and *E. coli* in TSB. Table 2 summarizes in vitro microbiological assay data.

**Table 2:** Minimum inhibitory concentration (MIC) of LyeTx I (control), LyeTx I-K-HYNIC and HYNIC-LyeTx I against *S. aureus* and *E. coli* in TSB

Peptide	*S. aureus* (ATCC® 6538)	*E. coli* (ATCC® 10536)
LyeTx I (non-modified peptide)	5.52 μmol^.^L^−1^	5.52 μmol^.^L^−1^
LyeTx I-K-HYNIC (C-terminal modified derivative)	5.05 μmol^.^L^−1^	10.10 μmol^.^L^−1^
HYNIC-LyeTx I (N-terminal modified derivative)	NI	NI

Values are expressed as median (*n* = 3). *NI* no inhibition

Previous data obtained for the non-modified peptide LyeTx I showed MIC’s of 3.79 μmol^.^L^−1^ and 7.81 μmol^.^L^−1^ for *S. aureus* and *E. coli*, respectively [8]. However, different assay conditions and other bacterial strains were employed, which can explain the slight differences in the MIC obtained in this study. The peptide LyeTx I-K-HYNIC derivative (C-terminal modification) maintained its antimicrobial activity, exhibiting similar MIC for *S. aureus* and about two-fold higher MIC for *E. coli*, when compared to the non-modified peptide. On the other hand, the peptide HYNIC-LyeTx I derivative (N-terminal modification) did not inhibit bacterial growth (NI: no inhibition). Thus, the N-terminal modification suppressed the antimicrobial activity of peptide HYNIC-LyeTx I derivative, suggesting that the N-terminal portion is important for the peptide interaction with bacteria. Therefore, only the peptide LyeTx I-K-HYNIC derivative was selected for further radiolabeling with ^99m^Tc, in order to be tested as a specific imaging probe for infectious.

### Radiolabeling and radiochemical purity of LyeTx I-K-HYNIC with ^99m^Tc

As the peptide HYNIC-LyeTx I derivative did not exhibit any antimicrobial activity, the radiolabeling process was performed only with the peptide LyeTx I-K-HYNIC derivative.

The radiolabeling procedure with ^99m^Tc can generate two main radiochemical impurities, ^99m^TcO_2_ and ^99m^TcO_4_^−^. High amounts of these entities may impair imaging data interpretation, once they accumulate in liver/spleen and in thyroid/stomach, respectively [18]. Therefore, it is important to determine and to optimize the radiochemical purity, which means the percentage of ^99m^Tc atoms that effectively bind the radiopharmaceutical molecules.

Herein, the radiolabeling of the peptide LyeTx I-K-HYNIC derivative with ^99m^Tc atoms was standardized taking into account some parameters: amount of peptide derivative, reducing agent, pH and heating (Table 3). ^99m^TcO_2_ molecules are retained at the point of application (R_f_ = 0.0) in both solvents, MEK and acetonitrile:water (1:1; v:v), once they form a colloid. In contrast, ^99m^TcO_4_^−^ migrates to the top of silica gel strip (R_f_ = 0.9–1.0) in both solvents. LyeTx I–K-HYNIC-^99m^Tc is a hydrophilic compound and thus it remains at the point of application when MEK is used as eluent and it migrates to the top of the silica gel strip with acetonitrile:water (1:1; v:v). Then, the later eluent was used to determine the amount of ^99m^TcO_2_, whereas the former was used to quantify ^99m^TcO_4_^−^.

**Table 3:** Radiolabeling standardization of the synthetic peptide LyeTx I-K-HYNIC derivative with ^99m^Tc

Amount of LyeTx I-K-HYNIC (SnCl_2_^.^2H_2_O = 200 μg; pH = 7; Δ = 15 min)	5 μg		10 μg		20 μg
RP (%)	73 ± 3^a^		83 ± 1^b^		82 ± 1^b^
Amount of SnCl_2_^.^2H_2_O (LyeTx I-K-HYNIC = 10 μg; pH = 7; Δ = 15 min)	100 μg		200 μg	250 μg	300 μg
RP (%)	82 ± 2		83 ± 1	87 ± 1	82 ± 3
^ 99m^TcO_2_ (%)	9 ± 1^a^		9 ± 1^a^	8 ± 0^a^	14 ± 2^b^
^ 99m^TcO_4_^−^ (%)	8 ± 1^a^		8 ± 0^a^	5 ± 1^b^	4 ± 1^b^
pH (LyeTx I-K-HYNIC = 10 μg; SnCl_2_^.^2H_2_O = 250 μg; Δ = 15 min)	5	6	7	8	9
RP (%)	77 ± 2^a^	88 ± 2^b^	87 ± 1^b^	83 ± 0^b^	72 ± 3^c^
Heating (100 °C) (LyeTx I-K-HYNIC = 10 μg; SnCl_2_^.^2H_2_O = 250 μg; pH = 7)	5 min		15 min	30 min	Unheated
RP (%)	71 ± 2^a^		87 ± 1^b^	81 ± 1^b^	57 ± 4^c^

Values are expressed as ‘mean ± SD’ (*n* = 3). Different letters indicate significant differences (*p* < 0.05). *RP* radiochemical purity

The radiochemical purity analysis (Table 3) showed that the radiolabeling procedure either with 10 or 20 μg of the peptide LyeTx I-K-HYNIC derivative presented a radiolabeling yield greater than that obtained when 5 μg of the peptide derivative was used. Then, the amount of 10 μg of the peptide LyeTx I-K-HYNIC derivative was selected for the next steps. Concerning to the reducing agent, SnCl_2_^.^2H_2_O, no significant differences in radiochemical purity was observed between the employed quantities. However, when 250 μg of SnCl_2_^.^2H_2_O was used, it was verified the lowest values of both impurities ^99m^TcO_2_ and ^99m^TcO_4_^−^. Thus, 250 μg of SnCl_2_^.^2H_2_O was selected in order to perform the other assays. Moreover, results showed that the optimal pH is between 6 and 8. Then, for in vivo experiments the pH = 7 was chosen. Finally, the radiolabeling process needed water bath heating (100 °C) for at least 15 min. As a result, the optimal radiolabeling procedure (10 μg of the peptide LyeTx I-K-HYNIC derivative; 250 μg of SnCl_2_^**.**^2H_2_O; pH = 7; heating for 15 min at 100 °C) yielded a radiochemical purity of 87 ± 1 % (*n* = 3) and the final preparation presented a specific activity of 37 MBq/mL.

### LyeTx I-K-HYNIC-^99m^Tc evaluation

**Fig. 4:**
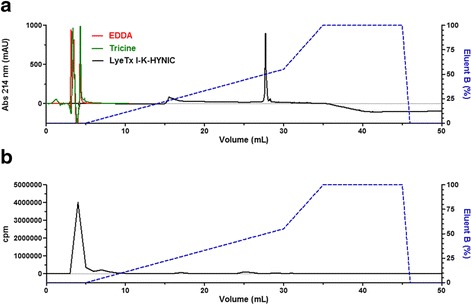
LyeTx I-K-HYNIC-^99m^Tc evaluation by RP-HPLC (717 Plus Autosampler, Waters, USA) associated with radioactivity determination of the collected fractions by automatic gamma counter (Wizard, Finland). **a** Chromatograms of EDDA (*red line*), tricine (*green line*) and LyeTx I-K-HYNIC (*black line*): ACE 5 C8 column (5 μm, 250.0 mm × 4.6 mm) equilibrated with 0.1 % (v:v) TFA in water (eluent A) and eluted by a linear gradient of 0.1 % (v:v) TFA in acetonitrile (eluent B); the flow was 1.0 mL^**.**^min^−1^ and the detection was at 214 nm. **b** Radiochromatogram of LyeTx I-K-HYNIC-^99m^Tc: ACE 5 C8 column (5 μm, 250.0 mm × 4.6 mm) equilibrated with 0.1 % (v:v) TFA in water (eluent A) and eluted by a linear gradient of 0.1 % (v:v) TFA in acetonitrile (eluent B); the flow was 1.0 mL^**.**^min^−1^. cpm: counts per minute

Besides radiochemical purity analysis, LyeTx I-K-HYNIC-^99m^Tc was evaluated by RP-HPLC in association with radioactivity measurement of the collected fractions (Fig. 4). First, the peptide LyeTx I-K-HYNIC derivative and the co-ligands (EDDA and tricine) were separately injected and detected at 214 nm (Fig. 4a). Afterwards, radiolabeled compound was injected and its fractions were collected. The radioactivity was measured in an automatic gamma counter (Fig. 4b) and the results revealed that the radioactivity was associated with the co-ligands, instead of the peptide LyeTx I-K-HYNIC derivative. These data indicate instability of the radiolabeled complex, suggesting ^99m^Tc transchelation from the peptide LyeTx I-K-HYNIC derivative to the co-ligands employed in this reaction. Although other authors had reported the importance of the co-ligands as stabilizing agents in the radiolabeling process, our findings did not show beneficial effects in this specific case [11, 19, 20]. Actually, it is related that EDDA is a strong chelating agent and, then, it might favor the transchelation, which is defined as the metal ^99m^Tc exchange from a weaker chelating agent to a stronger one [12, 21]. Therefore, further studies will be necessary to improve radiolabeling conditions in order to reach a better stability of LyeTx I-K-HYNIC-^99m^Tc.

## Conclusions

In summary, two peptide LyeTx I derivatives modified with the chelating agent HYNIC were synthesized, HYNIC-LyeTx I (N-terminal modification) and LyeTx I-K-HYNIC (C-terminal modification). The synthetic crude products were properly purified by RP-HPLC, as shown by MALDI-ToF-MS and RP-HPLC analyses. In vitro assay revealed that the attachment of HYNIC in the C-terminal portion of peptide LyeTx I did not compromise its antimicrobial activity and that the N-terminal portion is important for its interaction with bacteria. However, radiolabeling procedure conditions must be better investigated in order to optimize the process concerning to the binding between ^99m^Tc and the chelating agent HYNIC. Thus, this complex could be evaluated as a specific imaging agent to distinguish septic and aseptic inflammation.

## Abbreviations

LyeTx I: cationic peptide isolated from *Lycosa erythrognatha* venom
C-terminal: carboxyl-terminal
*E. coli*: *Escherichia coli*
*S. aureus*: *Staphylococcus aureus*
^99m^Tc: technetium-99m
^99^Mo/^99m^Tc generator: molibdenium-99/technetium-99m generator
HYNIC: 2-hydrazinonicotinamide
N-terminal: nitrogen-terminal
EDDA: ethylene diamine-N,N’-diacetic acid
TFA: trifluoroacetic acid
DMF: N,N-dimethylformamide
RP-HPLC: reverse phase-high performance liquid chromatography
ATCC: American type culture collection
HYNIC-LyeTx I: peptide LyeTx I derivative with the chelating agent HYNIC attached to its N-terminal residue
LyeTx I-K-HYNIC: peptide LyeTx I derivative with the chelating agent HYNIC attached to the lateral amino group of an extra lysine residue coupled to the C-terminal portion
Fmoc: N-9-fluorenylmethyloxycarbonyl
v:v: volume per volume
MALDI-ToF-MS: matrix-assisted laser desorption ionization time of flight mass spectrometer
MS: mass spectrometer
TSB: tryptic soy broth
MIC: minimum inhibitory concentration
NaCl: sodium chloride
w:v: weight per volume
SnCl_2_^**.**^2H_2_O: stannous chloride dehydrate
HCl: hydrochloric acid
Na^99m^TcO_4_: sodium pertechnetate
q.s. ad: quantity sufficient added
MEK: methyl ethyl ketone
^99m^TcO_4_^−^: free technetium
^99m^TcO_2_: hydrolyzed technetium
SD: standard deviation
ANOVA: analysis of variance
M_w_: molecular weight
CF: collected fraction
PP: peak of peptide
NI: no inhibition
RP: radiochemical purity
cpm: counts per minute
